# High Levels of Well‐Being and Being Infected With the COVID‐19 Virus Predicted Post‐Traumatic Growth in Healthcare Workers

**DOI:** 10.1111/jnu.70080

**Published:** 2026-04-08

**Authors:** Chiara Angelone, Giorgio Li Pira, Chiara Ruini

**Affiliations:** ^1^ Department of Psychology University of Bologna Bologna Italy; ^2^ Department of Life Quality Studies University of Bologna Rimini Italy

**Keywords:** COVID‐19, healthcare, positive mental health, post‐traumatic growth, psychological distress, well‐being

## Abstract

**Introduction:**

Healthcare workers (HCWs) are continuously exposed to stress and potentially traumatic experiences, as during the COVID‐19 pandemic. This research aims to investigate the correlates and predictors of Post‐traumatic growth (PTG), a positive outcome following adversity, in a group of HCWs during the COVID‐19 pandemic.

**Design:**

Cross‐sectional design.

**Methods:**

The sample included 168 HCWs (almost 43% were nurses working in hospitals or aging facilities) who were assessed with the PTG Inventory (PTGI) and other indicators of psychological distress (DASS‐21) and well‐being such as the Positive and Negative Affect Schedule (PANAS), the Mental Health Continuum Short‐Form (MHC‐SF), and the Satisfaction with Life Scale (SWLS). Regression analyses were calculated to evaluate the relationships among variables.

**Results:**

PTG Inventory positively correlated with SWLS (*r* = 0.256, *p* < 0.001) and MHC (*r* = 0.315, *p* < 0.001), but no correlations with anxiety and depression emerged. Female gender (*β* = 0.248, *p* = 0.001), COVID‐19 infection (*β* = 0.222, *p* = 0.003), and MHC Total score (*β* = 0.294, *p* = 0.008) predicted PTGI. Additionally, a significant curvilinear *U*‐shaped relationship existed between DASS‐stress and PTGI levels (*β* = 0.541, *p* = 0.021), meaning that PTG was lower at a medium level of stress.

**Conclusions:**

During the pandemic PTGI in HCWs was more directly predicted by well‐being indicators than distress. Prioritizing their well‐being, especially in times of crises, could aid in managing stress and trauma in healthcare settings.

## 1 Introduction

Worldwide the COVID‐19 Pandemic put a strain on healthcare workers (HCWs) who found themselves engaged in curing infected patients and in dealing with the inevitable overcrowding of the medical institutions. These stressful working conditions were found to be associated with increased rates of mental and physical exhaustion, sleep problems, burnout, anxiety, depression and post‐traumatic stress symptoms following the emergency (Elkholy et al. 2020; Johnson et al. 2020; Kang et al. 2020; Huang and Zhao 2021; Saragih et al. 2021). Nurses, young people, women and frontline workers were especially identified as being most at risk of developing mental health problems (Chong et al. 2023; Chutiyami et al. 2021; Tang et al. 2024). Among mental health problems, HCWs often find themselves indirectly exposed to the traumatic events their patients have experienced and may also incur vicarious trauma (VT; McCann and Pearlman 1990). Nonetheless, positive psychological trajectories were also observed in HCWs during and in the aftermath of the pandemic (Chutiyami et al. 2021; Kalaitzaki and Rovithis 2021; Tang et al. 2024), particularly when referring to the concept of *posttraumatic growth* (PTG; Calhoun and Tedeschi 1999, 2001). PTG is described as a positive improvement in functioning that individuals experience in various areas of their lives after facing trauma, and which goes beyond their pre‐trauma state (Tedeschi and Calhoun 2004). PTG was found in medical professionals as early as after the very first stages of the pandemic (Chen et al. 2021; Finstad et al. 2021). In fact, PTG, as well as stress, is often reported among HCWs who constantly interact with people affected with serious illnesses and injuries (Jurišova 2016; Oginska‐Bulik and Zadworna‐Cieslak 2018; Ogińska‐Bulik et al. 2021; Shakespeare‐Finch et al. 2003). Trauma indirect exposure is also known to positively affect professionals' well‐being and results in the phenomenon described as *Vicarious Posttraumatic Growth* (VPTG; Arnold et al. 2005; Doherty et al. 2020). A large body of research documented that professionals who work under traumatic circumstances and/or interact with trauma survivors often reported enhanced levels of growth in both their personal and professional lives (Dar and Iqbal 2020). They also show higher well‐being in dimensions such as life satisfaction, job satisfaction and sense of competence (Kim et al. 2016; Guhan and Liebling‐Kalifani 2011; Mostarac and Brajković 2022; Rodríguez‐Rey et al. 2019; Tsirimokou et al. 2022; Xie et al. 2022). A recent meta‐analysis published in this journal investigated PTG in nurses and has identified both personal (cognitive and emotional resources) and contextual factors (work‐system; event related characteristics) associated with PTG (Tang et al. 2024), highlighting the crucial interaction between nurses' and workplace well‐being.

According to Keyes' conceptualization, well‐being is an essential contributor for individuals' mental health. He postulated the *Mental Health Continuum* (MHC; 2002, 2005), where high levels of well‐being correspond with complete mental health (also known as the state of *flourishing*) characterized by positive feelings and good social and psychological functioning. Individuals on the “incomplete” side of the spectrum are categorized as *languishing* (i.e., more vulnerable for mental illnesses), whereas those whose mental health falls in between are referred to as having *moderate mental health*; thus presenting impairments in some dimensions of well‐being (Keyes 2002).

The correlation between PTG and flourishing mental health has been scarcely explored among HCWs. Nonetheless, it could be argued that positive mental health plays an important role in preventing HCWs from developing trauma‐related symptoms and in steering them towards a PTG trajectory. Conversely, being in a languishing state could increase their risks of developing clinically relevant post‐traumatic stress symptoms (Bassi et al. 2021). On the other hand, the relationship between PTG and negative indicators of mental health is still unclear, as current research presents mixed results. While some studies report trauma‐related stress to have a negative or null correlation with PTG (Hyun et al. 2021; Veronese et al. 2022), others suggest that the two may be positively related and coexist in trauma survivors (Shakespeare‐Finch and Lurie‐Beck 2014; Yeung et al. 2022). Given those heterogeneous results, some authors advanced the possibility that the relationship between posttraumatic growth and trauma‐related distress would be better explained as curvilinear (e.g., Dar and Iqbal 2020; Butler et al. 2005; Lechner et al. 2006; Shakespeare‐Finch and Lurie‐Beck 2014; Tsai et al. 2015). Thus, an increase in distress would then correspond to an increase in PTG, but when a traumatic event is especially challenging and the symptoms become extreme, PTG would instead decrease and the curvilinear relationship take a negative turn (McCaslin et al. 2009; Shakespeare‐Finch and Lurie‐Beck 2014; Yılmaz‐Karaman et al. 2022; Wu et al. 2009). As such, those who experience the highest growth would be those who report only an average amount of trauma‐related stress (Grubaugh and Resick 2007; Kleim and Ehlers 2009; Shakespeare‐Finch and Lurie‐Beck 2014; Thomas et al. 2021).

Identifying the factors that contribute to the development of PTG in HCWs is crucial for designing future interventions (Tang et al. 2024). In fact, posttraumatic growth is rarely an intentional goal and cannot be considered an expected outcome of trauma‐focused interventions. Therefore, identifying and targeting the elements that influence and predict higher levels of PTG in HCWs may be the most effective way to promote its development in this population.

## 2 The Present Study

This study aims to evaluate PTG, psychological distress and well‐being of a sample of HCWs who were directly and indirectly engaged in patient care during the COVID‐19 pandemic in Italy. Secondly, we sought to determine whether participants' levels of posttraumatic growth (PTG) were influenced by indicators of psychological distress and well‐being, such as positive affect, positive mental health and satisfaction with life on one hand, and depression, stress, anxiety and negative affect, on the other. Based on the literature reviewed above, we hypothesize that positive indicators of mental health and well‐being would predict and/or be directly correlated with higher levels of PTG. Regarding psychological distress, we expect to observe either a negative linear relationship with PTG (Hyun et al. 2021; Veronese et al. 2022), or a curvilinear relationship where PTG coexists with distress after the traumatic experience of COVID‐19.

## 3 Methods

### 3.1 Sample and Procedures

The present research is part of a larger research project on psychological well‐being, distress, burnout and resilience of Italian HCWs during the pandemic, that was partially published in previous articles by the same group of investigators (Ruini et al. 2023; Ruini et al. 2025). Participants were recruited by contacting healthcare professional associations/local boards and by contacting administrative officers/CEO, directors of hospitals and nursing homes in Northern Italy. The research aims and procedures were illustrated via mail to various hospitals' human resources divisions and advisory boards in the country. Following their approval of the study, a weblink and a QR code containing an online survey were created and distributed in different hospitals, healthcare organizations and institutions across Northern Italy. Data were collected between January 2021 and January 2022 on a voluntary basis and with full anonymity of participants (they were asked to log in with a numeric code). Before filling in the online survey participants had to provide their written informed consent to the study participation. Following a snowball sampling procedure, participants were also encouraged to share the QR code survey with other colleagues and co‐workers.

An “a priori” power analysis was conducted using G*Power to determine the required sample size for multiple linear regression models. We aimed to detect a medium effect size (*f* ^2^ = 0.15) with a power = 0.95 and an *α* level = 0.05. The analysis included 11 predictor variables. The calculation yielded a minimum required sample size of *N* = 119.

A total of 168 HCWs completed the survey.

### 3.2 Assessment

The assessment procedures are described in detail in another published article (Ruini et al. 2023). We collected data regarding age, gender, marital status, education, type of healthcare profession, job seniority, workplace, and direct contact with patients during the pandemic. Participants were also asked whether they had been infected with the COVID‐19 virus (yes/no).

*Psychological well‐being and positive mental health* were assessed using the following questionnaires:

*Post‐Traumatic Growth Inventory* (PTGI; Tedeschi and Calhoun 1996): The self‐report provides scores of 5 dimensions of post‐traumatic growth: a greater appreciation of life; a change in the sense of priorities; improved relationships with others; greater sense of personal strength, new possibilities for one's life, and spirituality. We used the short version of the inventory, which consists of 10 items. Participants indicate their degree of agreement with the content of each item, in a 6‐level Likert scale. The total score was calculated by adding up each item's score. The Cronbach's *α* is 0.959 in this sample.

*Positive and Negative Affect Schedule* (PANAS; Watson et al. 1988): The questionnaire assesses positive affect (PA, 10 items) and negative affect (NA, 10 items). The former subscale measures the degree to which a person feels enthusiastic, active, and determined; while the latter measures unpleasant emotional states, such as anger, guilt, and fear. Respondents had to indicate the frequency of positive or negative emotions on a 5‐point Likert scale. The Cronbach's *α* for this sample is 0.794 for PA and 0.932 for NA.

*Mental Health Continuum Short‐Form* (MHC‐SF; Keyes 2002): It evaluates the frequency of three domains of well‐being: emotional (3 items EWB), psychological (6 items PWB), and social well‐being (5 items SWB), according to a Likert scale from 0 ‘never’ to 5 ‘everyday’. The 14 items are then summed up to form a total well‐being score. In the present study, the Cronbach's *α* was 0.950.

*The Satisfaction with Life Scale* (SWLS; Diener et al. 1985): It measures the overall satisfaction with one's life using 5 items. Individuals indicated their degree of agreement with the content of the items using a 7‐point Likert scale. The 5 items are then summed up to form a total life satisfaction score. In the present study, the Cronbach's *α* was 0.925.

*Psychological Distress* was assessed using the following questionnaire:

*Depression Anxiety Stress Scales Short Version* (DASS‐21; Henry and Crawford 2005): It includes three subscales (depression, anxiety and stress), for a total of 21 items (7 items per subscale): Participants indicated their agreement using a Likert scale from 0 (did not apply to me at all) to 3 (applied to me very much). Scores are provided per each subscale and then by multiplying them by 2. The DASS‐total scale thus may range between 0 and 120, and the scores for each of the subscales may range between 0 and 42. In the present research, the Cronbach's *α* was 947 for the DASS‐21 total score.

### 3.3 Statistical Analyses

The normal distribution of the data was calculated with Skewness and kurtosis analyses, while bivariate correlations were calculated using Pearson's *r* value.

In order to evaluate the psychosocial correlates of posttraumatic growth, we applied a multivariate linear regression analysis. A three‐step regression model (method enter) was used, where sociodemographic data (age, gender, direct contact with patients during the pandemic, having been infected with the COVID‐19 virus) (step1), indicators of psychological distress (DASS Stress, Anxiety and Depression scales and PANAS Negative Affect scale) (step2), and indicators of well‐being (MHC Total Score, PANAS Positive Affect scale and SWLS Total score) (step3) were entered, considering PTGI Total Score as the dependent variable. Finally, to further explore the nature of the relationship between negative indicators of mental health and PTG, as in previous studies it resulted to be curvilinear, we performed a two‐step regression analysis (method enter) with squared indicators of psychological distress as the independent variable to predict PTGI Total Score (dependent variable).

The partial eta squared as a measure of effect size was used, with a value of 0.1 considered as a large effect, a value of 0.4 as a medium effect and a value of 0.01 as a small effect (Huberty 2002). The significance level was set at *p* < 0.05. Analyses were performed with the Statistical Package for the Social Sciences (SPSS), version 28.

## 4 Results

### 4.1 Sample Analysis

As described in other published articles (Ruini et al. 2023, 2025), a total of 168 HCWs completed the online survey: 137 females (81.5%) and 31 males (18.5%). The mean age was 43.68 years (SD = 10.741), ranging from 22 to 71 years. Most of the participants were nurses and midwives (23.7%), nurses working in aging facilities (19.1%), healthcare assistants (25.4%), physicians (8.7%), psychologists (6.9%), social workers, professionals in technical (e.g., technicians in radiology, biomedical lab) and rehabilitation areas (e.g., physiotherapists) (2.9%). Most of them (69.6%) worked in direct contact with patients (frontline workers: 94 females; mean age = 42.9 ± 11.56 years). Fifty‐one individuals (43 females; mean age = 45.48 ± 8.37 years) were second line workers (HCWs who work in healthcare facilities but were not required to interact with patients).

The 83.3% of participants (*N* = 140) reported to have never been infected with COVID‐19, whereas the remaining 16.7% (*N* = 28) attested to have tested positive to the COVID‐19 virus. The mean age of those who were never infected was 44.03 years (DS = 10.66), whereas those who had COVID‐19 infection had a mean age of 41.76 years (SD = 11.20).

### 4.2 Correlation Among Variables

The data distribution around PTGI Total Score was normal and symmetrical, with both skewness and kurtosis indexes ranging between +2 and −2 (Skew = 0.35, SE = 0.19; Kurt = −0.75, SE = 0.38). Table 1 illustrates the bivariate correlations between Posttraumatic growth and the other selected variables, representing psychological distress (stress, depression, anxiety, negative affect) and well‐being (satisfaction with life, positive affect, complete mental health) indicators. PTGI scores resulted to be positively related only to positive indicators of mental health, such as SWLS (*r* = 0.256, *p* ≤ 0.001) and MHC (*r* = 0.315, *p* ≤ 0.001) scores. However, no correlations could be found between PTGI and PANAS Positive Affect Scale, which were significantly related to all other variables except for PTGI. No significant correlation was detected between PTGI and indicators of psychological distress.

**TABLE 1:** Correlation matrix among variables in the total sample of Italian HCWs (*N* = 168).

	PTGI	DASS ANX	DASS DEP	DASS STRESS	PANAS NEG_AFF	PANAS POS_AFF	SWLS	MHC
PTGI	—	0.025	−0.099	−0.064	−0.038	0.129	0.**256** **	0.**315** **
DASS ANX			0.730**	0.633**	0.606**	−0.295**	−0.285**	−0.353**
DASS DEP			—	0.747**	0.664**	−0.436**	−0.457**	−0.503**
DASS STRESS				—	0.703**	−0.375**	−0.378**	−0.347**
PANAS NEG_AFF					—	−0.307**	−0.448**	−0.400**
PANAS POS_AFF						—	0.388**	0.616**
SWLS								0.591**
MHC								—

*Note:* The significance of bold value is represent ** *p* < 0.001.

Abbreviations: DASS ANX, DASS‐21 Anxiety Scale; DASS DEP, DASS‐21 Depression Scale; DASS STRESS, DASS‐21 Stress Scale; MHC, Mental Health Continuum; PANAS NEG AFF, PANAS Negative Affect Scale; PANAS POS AFF, PANAS Positive Affect Scale; PTGI, Posttraumatic Growth Inventory; SWLS, Satisfaction With Life Scale.

** *p* < 0.01.

### 4.3 Regression Analysis

A three‐step regression model (method enter) was calculated to predict posttraumatic growth (dependent variable). It revealed that variables included in the third model predicted 24.9% of the variance (*F* = 4.451; *p* < 0.001). Particularly, female gender (*β* = 0.248, *p* = 0.001), having been infected with the COVID‐19 virus (*β* = 0.222, *p* = 0.003) and Mental Health Continuum Total score (*β* = 0.294, *p* = 0.008) all significantly predicted Posttraumatic growth scores (see Table 2).

**TABLE 2:** Regression models predicting Posttraumatic growth in the total sample of Italian HCWs (*N* = 168).

	Model 1		Model 2		Model 3	
	*β*	*p*	*β*	*p*	*β*	*p*
Age	0.046	0.542	0.046	0.623	0.064	0.391
Gender (0 = M; 1 = F)	0.241	0.002	0.233	0.003	0.**248**	0.**001**
Frontline workers	0.063	0.405	0.078	0.310	0.110	0.137
COVID‐19 infection (0 = no; 1 = yes)	0.265	0.001	0.241	0.002	0.**222**	0.**003**
DASS ANX			0.119	0.313	0.083	0.466
DASS DEP			−0.200	0.153	0.004	0.977
DASS STRESS			−0.038	0.763	−0.089	0.461
PANAS NA			0.072	0.527	0.180	0.109
PANAS PA					−0.050	0.603
SWLS					0.179	0.056
MHC					0.**294**	0.**008**
*R* ^2^	0.125		0.142		0.249	
*R* ^2^ change	0.125		0.017		0.106	
*F* value	5.534	< 0.001	3.135	0.003	**4.451**	**< 0.001**

Abbreviations: DASS ANX, DASS‐21 Anxiety Scale; DASS DEP, DASS‐21 Depression Scale; DASS STRESS, DASS‐21 Stress Scale; MHC, Mental Health Continuum; PANAS NEG AFF, PANAS Negative Affect Scale; PANAS POS AFF, PANAS Positive Affect Scale; SWLS, Satisfaction With Life Scale.

### 4.4 Curvilinear Regression Analysis

**FIGURE 1:**
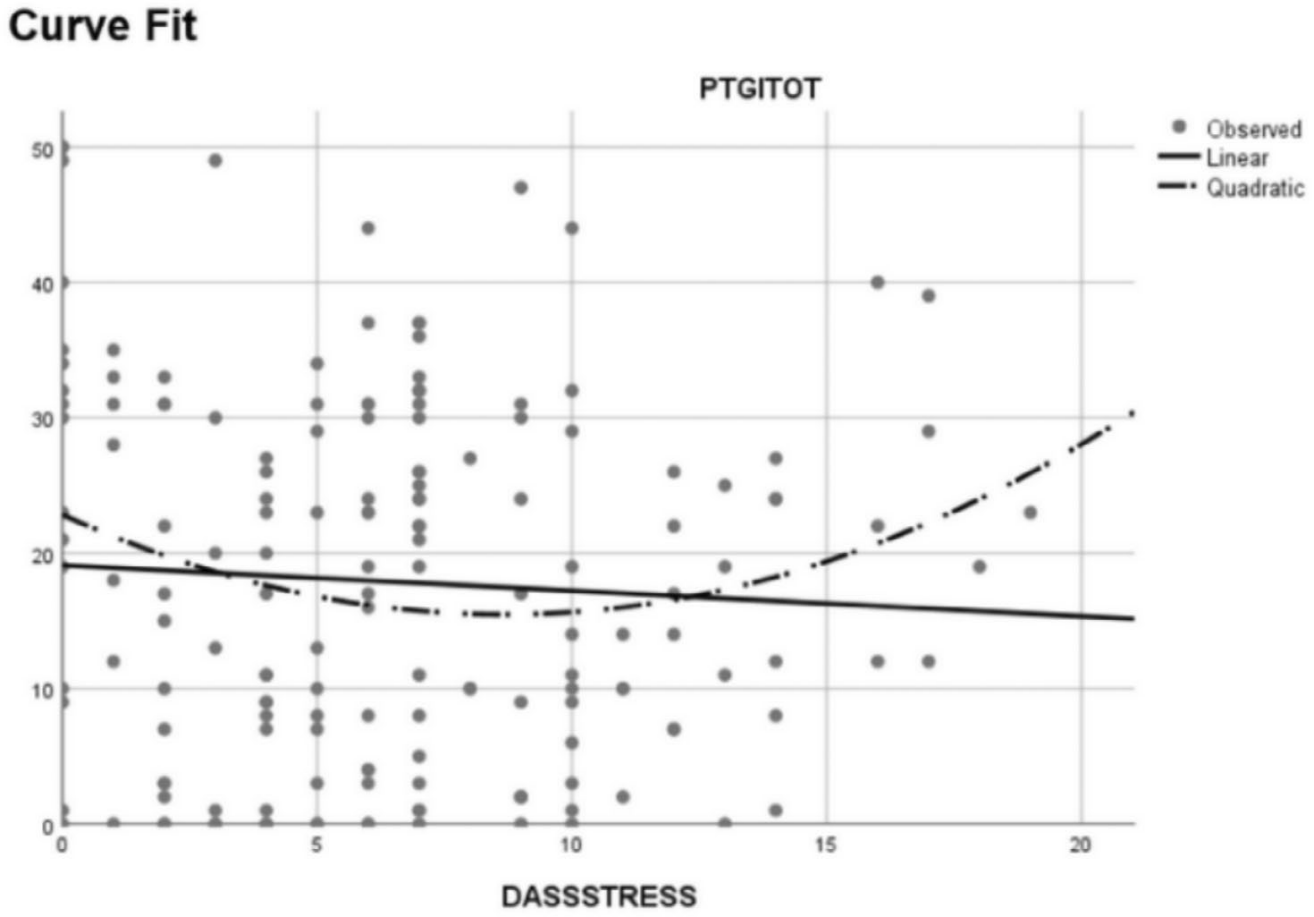
Quadratic and linear relation of PTGI scores to DASS stress scores.

The linear regression model with stress (DASS score) as independent variable and PTG as dependent variable did not result to be significant. However, once the squared variables were inserted in the second model, an *R* ^2^ change value of 0.33 was obtained for the stress subscale. This value was associated with an *F* change of 5.431 and resulted to be statistically significant (*p* = 0.021). The *β* of the squared variable resulted to be positive (*β* = 0.541, *p* = 0.021), indicating that a *U*‐shaped curve best described the relationship between stress and PTG in our sample (see Figure 1).

## 5 Discussion

The aim of this study was to evaluate PTG and its correlates/predictors in a sample of Italian HCWs during COVID‐19 pandemic, and to verify whether PTG levels were influenced by indicators of well‐being and psychological distress. PTGI scores resulted to be positively related to SWLS and MHC scores, indicating that HCWs who experienced PTG were also more likely to be satisfied with their lives and to have flourishing mental health. The literature on these associations confirms our findings. Positive associations between SWL and PTG have been detected across multiple samples, such as cancer survivors, people with physical disabilities and university students during the COVID‐19 lockdown (Kim et al. 2016; Mostarac and Brajković 2022; Xie et al. 2022). Other studies found a direct positive correlation between the constructs, with one even reporting the two to have a joint mitigating effect on COVID‐19 and trauma‐related stress (Veronese et al. 2017, 2022). In the case of HCWs population, some investigations have found positive correlations between PTG and satisfaction with life in nurses working in psychiatric and pediatric units who were exposed to work‐related stress and trauma‐inducing situations (Rodríguez‐Rey et al. 2019). These findings suggest that while—or after—facing potentially traumatic situations, such as the COVID‐19 pandemic, HCWs who report higher life satisfaction may also be more likely to experience PTG. Indeed, the very definition of PTG entails an increased appreciation of life in the aftermath of trauma (Tedeschi and Calhoun 1996; Tedeschi and Calhoun 2004).

In our sample, PTG was also correlated to positive mental health, as measured by the MHC total scores (Keyes 2005). Although the relationship between these constructs has been scarcely investigated in the literature so far, we can assume that flourishing mental health may serve as a protective factor in the face of stress and may lead HCWs to develop PTG, rather than psychopathology. Likewise, individuals who have experienced PTG might also have greater opportunities to achieve flourishing mental health (Middleton 2016). In fact, according to some authors (Joseph and Linley 2008), the experience of growth among trauma survivors can be compared to the development of higher levels of psychological well‐being. Furthermore, several studies have reported a significant positive correlation between posttraumatic growth, interpersonal relationships and social support (García et al. 2023; Joseph et al. 2012; Linley and Joseph 2004; Tedeschi and Calhoun 2004), which can be subsumed under the rubric of social wellbeing. We can therefore conclude that flourishing HCWs have more opportunities to develop PTG and a lower risk of being negatively affected by stressful conditions such as the COVID‐19 (Keyes et al. 2010). However, our results indicate no correlation between posttraumatic growth and the positive affect scale of the PANAS. This confirms a previous hypothesis (Joseph and Linley 2008) that argues that PTG does not entirely equate with experiencing positive emotions (i.e., hedonic well‐being). On the contrary, feelings of sadness and distress brought on by the trauma are usually reported to exist alongside PTG for a long time after the traumatic event has occurred (Tedeschi and Calhoun 1996). Rather, PTG seems to be associated with cognitive dimensions of wellbeing (i.e., satisfaction with one's life, purpose and meaning etc.), which indeed have influenced the level of PTG in our sample of HCWs.

Besides well‐being dimensions, our regression model showed that female gender and having been infected with COVID‐19 positively correlated to posttraumatic growth in our sample of medical practitioners. The literature written on the relation between gender and PTG is extensive and validates our findings. Two metanalyses highlighted that women are more likely to experience higher PTG than their male counterparts (Henson et al. 2020; Wu et al. 2019). Similarly, several studies were conducted on the relationship between gender and PTG in samples of HCWs and nursing students during the pandemic, all of which found that women presented significantly higher scores in the PTGI than men (Hamama‐Raz et al. 2020; Lin et al. 2022; Menculini et al. 2021; Sarıalioğlu et al. 2022; Yıldız 2021).

Our study found that having been infected with COVID‐19 was a significant correlate of posttraumatic growth, meaning that HCWs in our sample who tested positive at least once showed higher levels of PTG than those who were never infected with the virus. Findings regarding this relationship among HCWs during the pandemic are still scarce, making it difficult to advance definitive explanations. It is possible that being infected with COVID‐19 represented a traumatic event for some participants, and that the associated negative emotions and subsequent personal reappraisal of the illness may have led them to experience higher levels of PTG compared to colleagues who were not infected. Suffering from COVID‐19 symptoms may have also altered the perception of personal vulnerability/invulnerability in HCWs, and this could have had an impact on their PTG levels, probably because the questionnaire contains items investigating the issue of “personal strengths” (Mostarac and Brajković 2022). This interpretation is in line with a recent article that evaluated PTG in Spain during the COVID‐19 pandemic and documented that infected individuals had higher levels of both anxiety and PTG compared to those who did not report COVID‐19 infection, even when accounting for protective factors such as resilience and life purpose (Noriega et al. 2024). Authors concluded that the experience of actual illness (symptomatic COVID‐19) was associated with greater self‐reported growth, supporting the idea that direct health threat exposure can be linked to PTG beyond pandemic stressors alone (Noriega et al. 2024). However, this article was not specifically focused on HCWs and it is possible that contracting the COVID‐19 virus was perceived by them as an occupational accident in the workplace. A recent publication revealed that occupational accidents might promote PTG in workers who engage in deliberate rumination and seek social support in the aftermath (García et al. 2023). Unfortunately, we have not assessed rumination and social support in our sample, but it is possible that the COVID‐19 infection experienced by a small number of HCWs in our sample triggered a certain amount of personal distress that, in turn, promoted subsequent PTG. Interestingly, no correlation emerged between PTGI scores and working in the frontline. This finding is not supported by the existing literature, as healthcare professionals who worked in close contact with COVID‐19 patients have generally been reported to exhibit higher posttraumatic growth than those who did not work in the frontline (Chen et al. 2021; Finstad et al. 2021; Lee and Lee 2020; Lin et al. 2022; Mittermeier et al. 2023). Further investigations into the nature of this relationship are needed in the future.

Another finding worth commenting is the fact that we found no linear correlation between PTG levels and indicators of psychological distress. This result confirms Hyun et al. (2021), who found no correlation between stress and posttraumatic growth in healthcare professionals who worked during the MERS epidemic in South Korea. Additionally, another investigation (Veronese et al. 2022) documented that COVID‐19‐related stress was inversely related to PTG in a sample of HCWs. Nevertheless, findings on the relationship between PTG and trauma‐related symptoms of anxiety, stress, and depression are mixed, and some authors posited that experiences of growth and distress are likely to coexist after trauma. This pattern has been described as a curvilinear relationship (Shakespeare‐Finch and Lurie‐Beck 2014). Indeed, we found a curvilinear relationship between PTGI scores and the stress scale of the DASS. However, the *U*‐shaped curve representing this relationship differed from the one usually found in the literature (see Figure 1). Previous research found that the relationship between PTG and stress‐related symptoms seems to strengthen when symptoms are moderate and decrease when they become more pervasive, drawing an inverted *U*‐shaped pattern (Butler et al. 2005; Lechner et al. 2006; Shakespeare‐Finch and Lurie‐Beck 2014; Tsai et al. 2015). Conversely, in our study PTG was higher at lower and higher levels of stress (see Figure 1).

These results can be interpreted as further evidence of the complex relationship between stress‐related symptoms and PTG. We may hypothesize that some of the HCWs included in the study experienced PTG associated with a reduction in psychological distress (Cui et al. 2021; Finstad et al. 2021; Yim and Kim 2022). Conversely, other participants may have experienced PTG without necessarily overcoming the stress elicited by the pandemic, thereby reporting high levels of both. This phenomenon would be consistent with previous findings (e.g., Chen et al. 2021; García et al. 2016; Oginska‐Bulik and Zadworna‐Cieslak 2018) which suggest that stress‐related symptoms can coexist with PTG. This was recently reported in a sample of war refugees, whose levels of PTG co‐occurred with stress symptoms in a curvilinear, *U*‐shaped pattern (Kangaslampi et al. 2022). Authors concluded that refugees may exhibit intense distress due to the cognitive processing of trauma, which, at the same time, may facilitate PTG. Similarly, Landi et al. (2022), documented that during the COVID‐19 pandemic, the relationship between PTG and stress symptoms was moderated by psychological flexibility, but only in individuals reporting high levels of distress, as opposed to those with minimum distress, whose psychological flexibility was unrelated to PTG. Thus, different cognitive processes and personal resources might be implicated in the process of PTG, depending on the amount of distress experienced (Gurowiec et al. 2022).

These observations are in line with other recent investigations on HCWs that confirmed a curvilinear association between their levels of PTG and distress, but the patterns were different according to the specific type of healthcare profession (Dar and Iqbal 2020; Manning‐Jones et al. 2017; Shiri et al. 2008a, 2008b). For instance, the curvilinear relationship was weak or even disconfirmed among nurses and hospital rehabilitation workers, while it was significantly more robust in psychologists, surgeons, and physicians (Dar and Iqbal 2020). These differences might be linked to the type of healthcare assistance provided. As a matter of fact, our sample was quite heterogeneous in terms of healthcare professions, and this might explain the unusual *U*‐shaped relationship between PTG and distress that emerged in our regression model (Figure 1).

An alternative explanation could be that, as a coping strategy during the pandemic, some HCWs may have reported *illusory*, rather than genuine growth (Shakespeare‐Finch and Lurie‐Beck 2014). In fact, individuals' self‐reports of growth sometimes reflect specific cognitive biases or positive reinterpretations of their attempts to cope with the traumatic experiences rather than actual psychological change (Coyne and Tennen 2010; Ruini 2017; Tang et al. 2024). In our sample, the lowest levels of PTG were observed among participants with moderate levels of stress—a finding that, to our knowledge, has not been reported in the literature. We may assume that the peculiarity of this population and the unique context of the pandemic may be linked to this unexpected finding. Future studies are needed to replicate these results and further elucidate this pattern of correlations in HCW.

## 6 Limitations

This study is limited by its preliminary nature, the self‐selected, homogenous sample, and the use of self‐reports as assessment methodology. This leaves the possibility of self‐report bias to inflate some of the significant correlations and it limits the external validity of the findings. Moreover, important variables such as the severity of COVID‐19 illness experienced by some participants, perceived social support, the characteristics of their workplaces and workload were not investigated in our study. They could have given us more information about the effects of experiencing COVID‐19 symptoms or working frontline vs. second line on their PTG levels. Finally, the cross‐sectional study design precluded any definitive conclusions about causality among variables. Follow‐up and longitudinal studies should be performed to investigate PTG, positive mental health and their relationships with psychological distress in HCWs.

## 7 Conclusions

This study underlines the complex relationship between PTG, well‐being and psychological distress in HCWs. In this population, PTG was significantly correlated to female gender, the level of flourishing mental health and to the infection with COVID‐19. Unexpectedly, psychological distress showed a *U*‐shaped curvilinear relationship with PTG, wherein participants with moderate levels of distress displayed the lowest level of PTG. A recent review found that PTG in healthcare settings is a peculiar and still understudied phenomenon, which could be explained by the interplay of personal systems, workplace systems and event‐specific characteristics (Tang et al. 2024). The findings of this investigation are consistent with these observations and highlight the need to develop intervention policies aimed at supporting the mental health of nurses and other healthcare professionals. Given that HCWs typically work in challenging and stressful conditions and are frequently exposed to trauma, it is essential to devote greater attention to the subject of PTG and to the factors that facilitate its spontaneous development. In our sample, HCWs who experienced post‐traumatic growth were more likely to report higher life satisfaction and better mental health. This underscores the importance of protecting and promoting the well‐being of HCWs, especially during periods of crisis or high stress. Our findings suggest that mental health and well‐being may act as protective factors in the face of potential trauma and may promote post‐traumatic growth. If confirmed by future research, intervention strategies focused on promoting well‐being and positive functioning could serve as valuable tools for fostering healthy and resilient healthcare organizations.

## 8 Clinical Resources

– PTG and COVID‐19: https://greatergood.berkeley.edu/article/item/can_any_good_come_of_our_covid_lockdowns.
– International Positive Psychology Association: Health & Well‐being Division: https://ippanetwork.org/ippa‐divisions/positive‐health‐and‐wellbeing/.
– ANA Nursing Resources Hub—Three Steps to Build Resilience: https://www.nursingworld.org/content‐hub/resources/nursing‐leadership/three‐steps‐to‐build‐resilience/.

## Funding

The authors have nothing to report.

## Ethics Statement

Since this study implied full anonymity and a voluntarily participation, without directly interacting with individuals, ethical approval was provided by the advisory boards of the healthcare institutions included in the study.

## Conflicts of Interest

The authors declare no conflicts of interest.

## Data Availability

Data are available upon request to the Authors.
